# A novel cryo-embedding method for in-depth analysis of craniofacial mini pig bone specimens

**DOI:** 10.1038/s41598-020-76336-3

**Published:** 2020-11-11

**Authors:** Pavla Ticha, Igor Pilawski, Xue Yuan, Jie Pan, Ustun S. Tulu, Benjamin R. Coyac, Waldemar Hoffmann, Jill A. Helms

**Affiliations:** 1 Division of Plastic and Reconstructive Surgery, Department of Surgery, Stanford University School of Medicine, 1651 Page Mill Road, Palo Alto, CA 94304 USA; 2 Clinic of Plastic Surgery, Third Medical Faculty, Charles University, Prague, Czech Republic; 3 Nobel Biocare Services AG, Zurich, Switzerland

**Keywords:** Dentistry, Preclinical research, Bone

## Abstract

The disconnect between preclinical and clinical results underscores the imperative for establishing good animal models, then gleaning all available data on efficacy, safety, and potential toxicities associated with a device or drug. Mini pigs are a commonly used animal model for testing orthopedic and dental devices because their skeletons are large enough to accommodate human-sized implants. The challenge comes with the analyses of their hard tissues: current methods are time-consuming, destructive, and largely limited to histological observations made from the analysis of very few tissue sections. We developed and employed cryo-based methods that preserved the microarchitecture and the cellular/molecular integrity of mini pig hard tissues, then demonstrated that the results of these histological, histochemical, immunohistochemical, and dynamic histomorphometric analyses e.g., mineral apposition rates were comparable with similar data from preclinical rodent models. Thus, the ability to assess static and dynamic bone states increases the translational value of mini pig and other large animal model studies. In sum, this method represents logical means to minimize the number of animals in a study while simultaneously maximizing the amount of information collected from each specimen.

## Introduction

Prior to their use in humans, medical devices are first evaluated in animal models (reviewed in^1,2^). Although in silico and in vitro models are gaining in popularity because of the ability to control variables ranging from physical stimuli to growth factor exposure^3^, animal models still remain the gold standard.

An ideal animal model should (1) exhibit a genome that is similar to humans; (2) the tissue/organ should bear an anatomy and physiology similar to humans; (3) the pathological response(s) of that tissue/organ should be similar to humans; and (4) the underlying mechanism(s) of a drug or devices’ actions should be representative of a human response to the same treatment. Most exploratory and developmental research uses rodent models because their physiology and pathophysiology are similar to humans, the tissues are amenable to histochemical, immunohistochemical and genetic analyses, and compared to large animals, are inexpensive to house^4–6^. Pivotal preclinical studies typically involve the use of large animal models because the size of the animal’s tissues and organs permit the testing of devices in their final, human-sized scale. For example, intravascular stents or ventricular assist devices can only be tested in animals with blood vessels and hearts that are anatomically comparable in size to those of humans^7^. Likewise, clinical relevance and efficacy of dental implants are tested in animals whose jaws are similar to human size (ISO 7405:2018;^8^). Here, we focused on one of the most commonly employed animal models for assessing dental implant function e.g., the mini pig (reviewed in^15^).

In the majority of mini pig studies, non-invasive imaging is typically carried out, along with some histologic examination^9–14^ but very few biomechanical, histochemical, or immunohistochemical assays are routinely performed. Consequently, relatively little detailed data can be gleaned about how, for example, an implant system affects osteocyte apoptosis and bone remodeling, the inflammatory response, or the advent and/or rate of new bone deposition in the peri-implant environment.

We view this paucity of data from large animal studies as a tractable problem. Our specific objective here was to develop techniques for the evaluation of mini pig craniofacial bone tissues that were on par with the tools and technologies that are readily available for rodent animal studies. We also sought to carry out these analyses in a time frame that made them practical for pivotal preclinical studies, where the duration of a study becomes a critical variable in moving a therapy into clinical practice.

We concentrated our analyses on bone, because current methods for hard tissue preparation are the most lengthy and complicated. We describe a relatively straightforward method for cryo-embedding and -sectioning craniofacial bone tissues from adult mini pigs. We provide details of each step of the procedure, histologic, histochemical and immunohistochemical analyses and mineral bone apposition measurements of the resulting tissues. By rendering mini pig tissues amenable to histochemical/immunohistochemical analyses, on par with that typically found in rodent studies, we open the possibility of gaining more insights from preclinical large animal studies. In turn, this may allow a more rigorous assessment of drugs and devices intended for use in humans.

## Methods

### Animals

8 miniature Yucatan pigs (Sus Scrofa Domesticus) skeletally mature females and castrated males supplied by Triporc/Sinclair were used for this study and provided to us via our collaboration partners (Nobel Biocare, Zürich, Switzerland). Mini pigs were 26–34 months of age at euthanasia and their weight was considered commensurate with their age e.g., ~ 90 kg. All protocols were reviewed and approved by the Comité institutionnel de protection des animaux d’AccelLAB (the Testing Facility’s Institutional Animal Care and Use Committee (IACUC)). The review insured compliance with Canadian Council on Animal Care regulations. All experiments were performed in accordance with relevant guidelines and regulations. Animals were housed in a contract research organization AccelLab, Quebec, Canada and it's Testing Facility was accredited by the Association for Assessment and Accreditation of Laboratory Animal Care (AAALAC). Details regarding mini pig handling are described in Supplementary Information.

Vital dye labeling data was obtained from mice and rats that were part of a separate study. The care and housing and experimental protocol for dynamic histomorphometry followed ARRIVE guidelines and were approved by the Stanford Committee on Animal Research (#13146).

### Vital dye labeling in mini pigs

Two vital dyes were employed, i.e., calcein green (10 mg/kg, dissolved in 0.15 M NaCl and 2% NaHCO_3_) and alizarin red (25 mg/kg, dissolved in saline solution). In brief, mini pigs received the fluorochromes by IV administration. Dyes were delivered into lateral auricular vein via a portable pump over the course of 30–60 min. The time interval between calcein green and alizarin red administration was 11 days. Vital dye labeling in mice and rats is described in Supplementary Information.

### Mini pig tissue processing

Details regarding sacrifice, tissue collection and fixation are described in Supplementary Information. Fixed mini pig hemi-mandibles were separated into ~ 5 cm^3^ portions using either a rotary tool outfitted with 40 mm diamond cutting discs or a low speed rotating saw to facilitate cryo-embedding media infiltration and tissue cryo-sectioning. All dissecting was performed under continuous irrigation with phosphate-buffered saline (PBS). Parts of each specimen were left in an undecalcified state and cryo-embedded, while other parts were decalcified and paraffin-embedded. This approach allowed further direct comparisons between the tissue handling methods.

### Cryo-preservation and infiltration

To cryo-preserve tissues, each specimen was maintained on a rocking platform at 4 °C in 50 mL of a 30% sucrose solution for 8 days. The sucrose solution was exchanged every 48 h for a total of 3 exchanges. Specimens were then infiltrated with mixture of polyethylene oxide (PEO) and carboxymethyl cellulose (CMC) (#C-EM001, Section lab, Hiroshima, Japan). Gradual infiltration of the viscous medium was achieved through stepwise dilution of PEO-CMC in the sucrose solution, e.g., in step 1, the 30% sucrose solution was diluted 1:1 with PEO-CMC; specimens remained in 15 mL of this solution for 48 h with slow shaking. In step 2, the solution consisted of 1:2.5 sucrose:PEO-CMC. In step 3, the solution consisted of 1:4 sucrose:PEO-CMC. In step 5, the solution was comprised of PEO-CMC only. Each step was undertaken at 4 °C, in a minimum of 15 mL, for 48 h with continuous slow shaking (Table 1).

**Table 1:** Tissue cryo-preservation timeline.

Solution	Duration	Conditions
4% PFA	72 h	4 °C
PBS or 70% ethanol	As needed	4 °C
30% sucrose	48 h	4 °C, slow shaking
30% sucrose	48 h	4 °C, slow shaking
30% sucrose	48 h	4 °C, slow shaking
30% sucrose	48 h	4 °C, slow shaking
30% sucrose:PEO-CMC (1:1)	48 h	4 °C, slow shaking
30% sucrose:PEO-CMC (1:2.5)	48 h	4 °C, slow shaking
30% sucrose:PEO-CMC (1:4)	48 h	4 °C, slow shaking
PEO-CMC	48 h	4 °C, slow shaking

### Cryo-embedding

**Figure 1:**
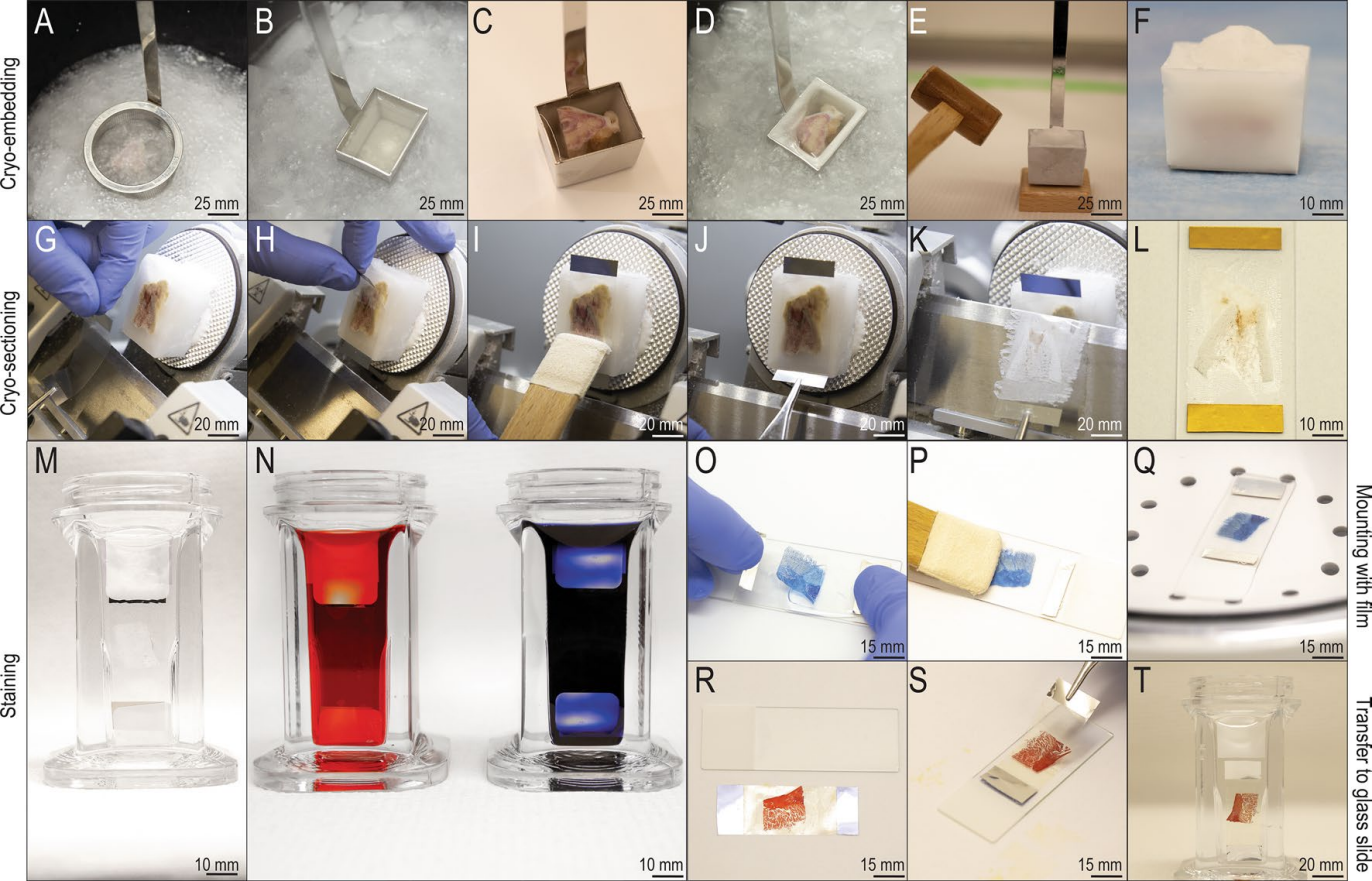
Cryo-processing allowed to yield hundreds of high-quality large tissue sections. (**A**) Cryo-preserved tissue specimens were frozen in hexane-dry ice coolant. (**B**) PEO-CMC in a container was cooled in the hexane-dry ice coolant until partially frozen. (**C**) The frozen specimen was transferred into the container with partly frozen PEO-CMC. (**D**) The container was immersed in the hexane-dry ice coolant. (**E**,**F**) A frozen specimen block released from its container. (**G**) Cryosections were collected on an adhesive film, (**H**) positioned over the surface and (I) rubbed against the specimen block. (**J**,**K**) During sectioning, the cryofilm was held with tweezers. (**L**) The flexible cryofilm was supported by a glass slide. (**M**,**N**) Tissue sections that had been collected on cryofilm were fixed in 4% PFA and stained. (**O**) Tissue sections that were affixed to cryofilm were then inverted onto glass slides; (P) the cryofilm was smoothed; and (**Q**) the glass slides were placed into a vacuum chamber. (**R**) To permanently transfer the tissue sections to a new glass slide, the supporting glass slide was removed and (**S**) the tissue section was inverted onto a silane-coated glass slide. (**T**) The adhesive film was dissolved in hexane or trichlorethylene, leaving behind the tissue section on the slide.

Once tissues were cryo-preserved and infiltrated with PEO-CMC, they were immersed in a hexane-dry ice mixture where dry ice was placed in a 5 L plastic beaker to which 500 mL of hexane was added. The cryo-preserved and infiltrated tissue specimen (~ 5 cm^3^) was positioned in a steel strainer and submerged into the hexane-dry ice coolant for 2–10 s until the surface of the specimen visibly appeared to be frozen (Fig. 1A). Care was taken to not over-freeze the specimen in order to prevent fissures in the tissue. In a separate stainless-steel container (#C-EC005, 3.5 cm × 2.5 cm, Section lab, Hiroshima, Japan), additional PEO-CMC was cooled in the hexane-dry ice coolant until partially frozen (Fig. 1B). The frozen mini pig specimen was then transferred into the partly frozen PEO-CMC with the sectioning plane oriented upwards (Fig. 1C); additional PEO-CMC was then applied to completely cover the tissue block. The container was returned to the hexane-dry ice coolant, this time fully immersed for 2–10 s (Fig. 1D). Specimen was visually inspected for the presence of fissures (a sign of over-freezing) and opacity (the more transparent the PEO-CMC, the less it was frozen). The specimen was re-immersed into the dry ice-hexane at 2–10 s intervals, with visual inspections after each dip, for a total of 2–5 min, until it was frozen completely. If fissures were evident, specimen was thawed, and the embedding/freezing process was repeated. Once fully frozen with no evidence of fissures, the specimen block was placed on a wooden stage and gently but firmly tapped with a wooden hammer to be released from the stainless-steel container (Fig. 1E). In this condition, specimen blocks could then be stored at − 20 °C or directly transferred to a cryostat for sectioning (Fig. 1F).

### Cryo-trimming, cryo-sectioning, and collection of tissue sections

To prepare for cryo-sectioning, the specimen block was attached to the chuck and secured in place with small amount of PEO-CMC. The specimen and chuck were then positioned in the cryostat chamber, which was maintained at − 24 °C. After a 20 min equilibration period, cryo-trimming was performed to eliminate extraneous tissues and to reach the region of interest (ROI) comprised of alveolar bone. Cryo-trimming was accomplished using a C-profile steel blade (#14021607116, Leica, Wetzlar, Germany). This blade was sufficiently firm and stable to allow trimming of the block however, it was not sufficiently firm and/or stable to permit high-quality cryo-sectioning. Rather, cryo-sectioning itself was performed using a D-profile tungsten carbide knife (#152460 M, Thermo Fisher Scientific, Waltham, MA, USA) positioned in the cryostat equipped with steel knife carrier (#705950, Steel knife carrier, Thermo Fisher Scientific, Waltham, MA, USA). In our hands, the D-profile tungsten carbide knife provided greater stability and firmness than other knives and was essential for the generation of high-quality mini pig hard tissue sections. Cryo-sectioning was initiated by generating 10–20 sections that were 1–12 µm; this allowed safe and gentle repositioning of the specimen prior to arriving at the ROI. Sectioning was then performed at 12 µm/section.

Cryosections were collected on an adhesive film (Fig. 1G). This flexible, transparent, non-fluorescent material consists of synthetic adhesive cryoglue compound of acrylic resin applied to a polyvinylidene chloride film that is 10 µm thick. Two types of adhesive film were employed. Cryofilm (#C-MK001-C2, cryofilm type 2C(9) 3.5 cm, Section lab, Hiroshima, Japan) allowed further straightforward processing and the majority of analyses of tissue sections affixed to the adhesive film. However, when visualized under polarized light, the adhesive film affected the polarization. Transfer film (#C-FT005, transfer film 3.5 cm, Section lab, Hiroshima, Japan) enabled transfer of the tissue section from the adhesive film to a glass slide which enabled further visualization of collagen organization using Picrosirius red staining.

One of these two adhesive films was positioned over the surface of a specimen block using cold tweezers, with the adhesive surface facing the specimen (Fig. 1H). The adhesive was gently rubbed against the specimen with a soft deerskin fitting tool (Fig. 1I). During cutting of 12 µm sections, the cryofilm was held taut with tweezers (Fig. 1J,K) and pulled gently to prevent crumpling of film or folding of tissue. Motorized sectioning mode with constant low speed 1–5 mm/s was applied in order to produce precise tissue sections. Adhesive film with affixed section was underlaid by supporting glass slide with section oriented upwards. (Fig. 1L).

In sectioning portions of the specimen which were particularly dense or where the embedding medium didn't fully infiltrate the tissue, fractures or crumbling occasionally occurred. Two strategies were employed in an attempt to overcome these problems: first, a few (~ 10–50) very thin (~ 1–2 µm) sections were cut and discarded; this strategy, however, was problematic if a crucial ROI was encountered. A second approach involved interrupting the sectioning, defrosting the specimen, and re-incubating it in 15 mL of PEO-CMC at 4 °C with continuous slow shaking. After 24 h, the specimen was prepared again for sectioning.

### Vital dye detection, image acquisition and quantification

Please see Supplementary Information.

### Statistical analyses

Statistical analyses are described in Supplementary Information.

### Histological staining of undecalcified, cryo-embedded tissue sections

Prior to histological staining, samples on cryofilm or transfer film were washed in PBS for 10 s, then immersed in 4% paraformaldehyde (PFA) for 20 s and rinsed in PBS (Fig. 1M). Thereafter, histological stainings were performed as described in Supplementary Information (Fig. 1N).

### Transfer and mounting of cryo-sectioned tissues

To mount tissue sections that had been affixed to cryofilm, the supporting glass slide was removed and discarded; a second, silane-coated glass slide (#7801, Lab Scientific, Highlands, NJ, USA) was prepared, to which ~ 1 mL of water-based mounting medium was applied. The tissue section on cryofilm was then inverted onto the mounting medium-covered glass slide (Fig. 1O). Excess mounting medium was removed from the glass slide using filter paper. Since the texture of the cryofilm tended to trap air bubbles, care was taken to keep the cryofilm smooth (Fig. 1P). Then slides were placed into a vacuum chamber for ~ 1 h (Fig. 1Q). Thereafter, slides were maintained at − 20 °C to avoid drying, which impaired quality.

To mount tissue sections affixed to transfer film, a different process was employed (Fig. 1R–T). Tissue sections on transfer film had the supporting glass slide removed and a silane-coated glass slide was prepared (Fig. 1R). In the absence of mounting medium the transfer film was inverted and positioned over the glass slide, sandwiching the tissue section between the transfer film and the glass slide (Fig. 1S). The adhesive was dissolved during an incubation period at room temperature in various solutions (Fig. 1T). For example, in a hexane solvent, the film loosened after 2–8 h. In a trichlorethylene solvent, the film loosened after 30–60 min. In both cases, the transfer film was gently removed. The tissue sections remained on the glass slide and, thereafter, tissue sections were cover-slipped with xylene-based mounting medium.

### Histochemistry and immunohistochemistry of undecalcified, cryo-embedded tissue sections

Please see Supplementary Information.

### Image acquisition of undecalcified, cryo-embedded, stained tissue sections

Please see Supplementary Information.

### Paraffin-processing of mini pig tissues

Please see details in Supplementary Information.

## Results

### A rapid method for generating undecalcified, cryo-embedded hard tissues

**Figure 2:**
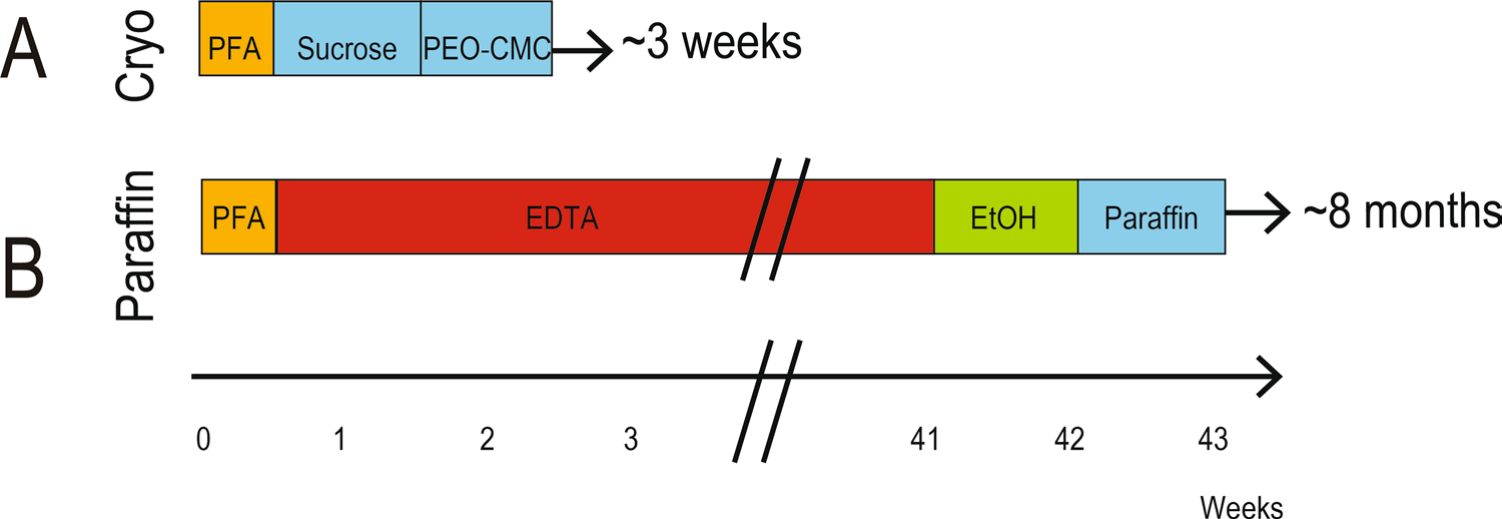
Cryo-processing method is relatively rapid. (**A**) Some blocks of craniofacial tissues from mini pigs were left in an undecalcified state and cryo-embedded while (**B**) other blocks from the same mini pig were decalcified and paraffin-embedded. *EDTA* ethylenediaminetetra-acetic acid, *EtOH* ethanol, *PFA* paraformaldehyde, *PEO-CMC* polyethylene oxide and carboxymethyl cellulose.

To determine whether hard tissues could be analyzed as thoroughly but much more rapidly using cryo-processing techniques as opposed to traditional paraffin methods, we undertook parallel studies. Craniofacial tissues from mini pigs were harvested and either left in an undecalcified state and cryo-embedded, or they were subjected to decalcification and paraffin-embedded (Fig. 2). For both methods, the time in fixation solution was equivalent (Table 1). Undecalcified samples were subsequently cryo-preserved in sucrose for ~ 1 week then infiltrated with PEO-CMC for ~ 1 week (Fig. 2A), followed by sectioning and staining. The total length of time from tissue harvest to sectioned samples was 3 weeks. In contrast, paraffin-embedded samples first had to be decalcified. EDTA decalcification took ~ 7 months. Following removal of calcium from the hard tissues, blocks were dehydrated using a graded ethanol series in paraffin for ~ 1 week. Afterwards they were infiltrated with paraffin at 60 °C in a vacuum chamber (~ 1 week) followed by paraffin-embedding. In total, tissues were ready for sectioning ~ 8 months after initiation of this process (Fig. 2B).

### Undecalcified, cryo-embedded and decalcified, paraffin-embedded tissues have a similar histologic appearance

**Figure 3:**
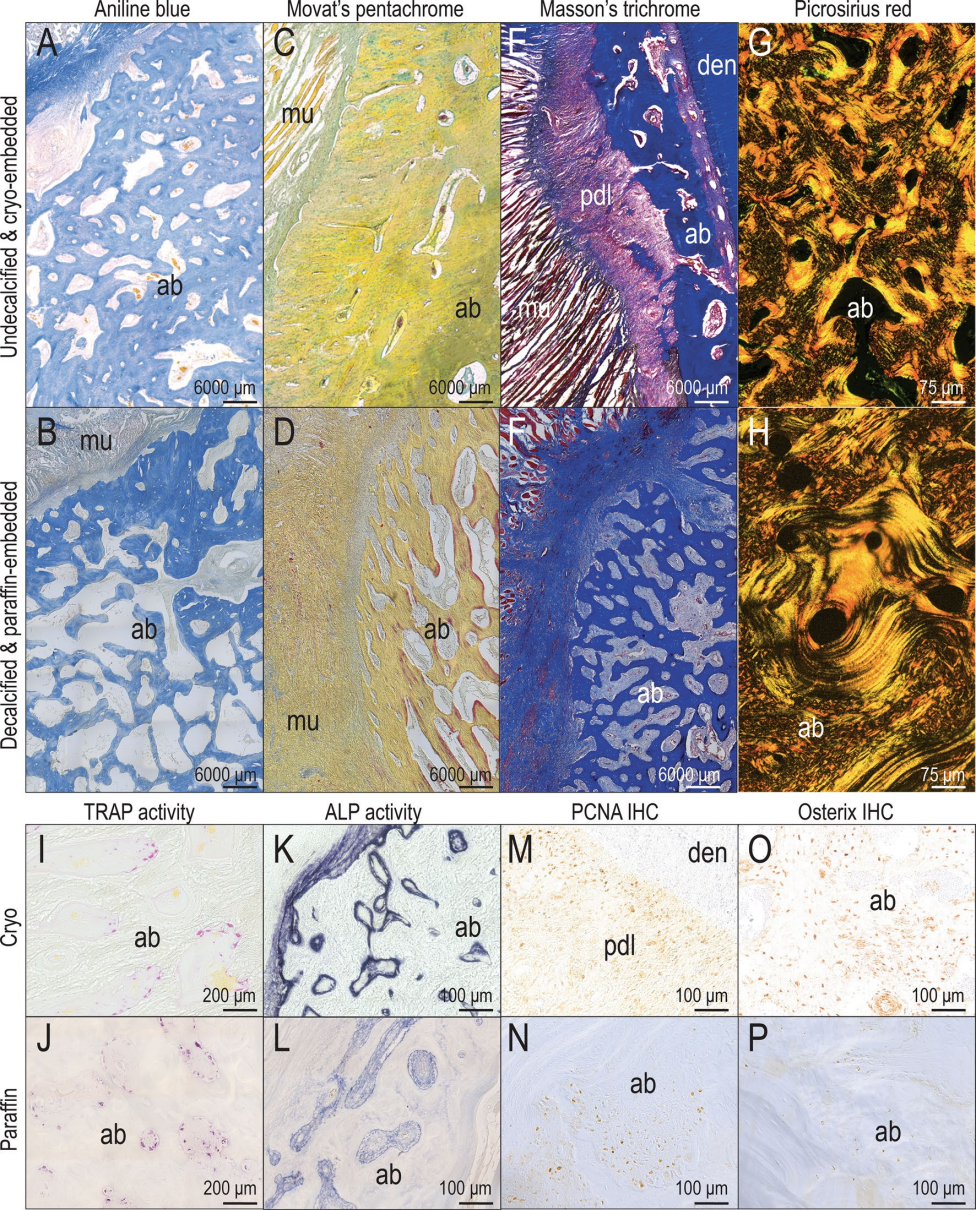
Cryo-processing of alveolar bone enables histological analyses undistinguishable from paraffin and histochemical and immunohistochemical analyses superior to paraffin-processing. (**A**) Cryo-embedding, coronal section on cryofilm, Aniline blue staining; (**B**) Paraffin-embedding, coronal section, Aniline blue staining; (**C**) Cryo-embedding, coronal section on cryofilm, Movat's pentachrome staining; (**D**) Paraffin-embedding, coronal section, Movat's pentachrome staining; (**E**) Cryo-embedding, coronal section on cryofilm, Masson's trichrome staining; (**F**) Paraffin-embedding, coronal section, Masson's trichrome staining; (**G**) Cryo-embedding, coronal section transferred to glass slide; Picrosirius red staining; (**H**) Paraffin-embedding, coronal section, Picrosirius red staining; (**I**) Cryo-embedding, transversal section on cryofilm, TRAP staining; (**J**) Paraffin-embedding, coronal section, TRAP staining; (**K**) Cryo-embedding, transversal section on cryofilm, ALP staining; (**L**) Paraffin-embedding, transversal section, ALP staining; (**M**) Cryo-embedding, transversal section on cryofilm, PCNA immunostaining; (**N**) Paraffin-embedding, transversal section, PCNA immunostaining; (**O**) Cryo-embedding, transversal section on cryofilm; Osterix immunostaining; (**P**) Paraffin-embedding, transversal section; Osterix immunostaining. Scale bars as indicated. Abbreviations: ab: alveolar bone, den: dentin, mu: muscle, pdl: periodontal ligament.

Given the rapid turnaround that cryo-processing allowed, we considered whether tissue integrity was preserved as well as in paraffin-embedded samples. We found in side-by-side comparisons that Aniline blue stained cryo-embedded tissues were indistinguishable from decalcified, paraffin-embedded samples (Fig. 3A,B). Image sharpness was lower in cryo-embedded tissues because undecalcified tissues had to be photographed through the cryofilm (compare Fig. 3A,B) but using Movat’s pentachrome staining to delineate mature osteoid matrix, there was no obvious difference in histologic appearance of the undecalcified, cryo-embedded samples compared to the decalcified, paraffin-embedded samples (Fig. 3C,D). Undecalcified, cryo-embedded tissues stained with Masson’s trichrome showed a striking delineation between muscle (red), the fibrous periodontal ligament (magenta), and the osteoid matrix (blue; Fig. 3E) whereas decalcified, paraffin-embedded samples had a more uniform coloration (Fig. 3F). Last, we used Picrosirius red staining to analyze collagen matrix organization; when viewed under polarized light, the cryo- and paraffin-processed tissues were indistinguishable: both highlighted the intermixed lamellar structure and characteristic basket-weave patterns in alveolar bone (Fig. 3G,H). From these histologic analyses we concluded that undecalcified, cryo-embedded and decalcified, paraffin-embedded craniofacial tissues had a similar appearance, although when brightfield microscopy was employed the presence of cryofilm led to a lower clarity of image of the acquired regions. Under polarized light, however, the two methods of tissue preparation resulted in qualitatively equivalent images.

### Enzymatic activity is preserved in undecalcified, cryo-embedded tissues

Undecalcified, cryo-embedded and decalcified, paraffin-embedded tissues from the same regions of the craniofacial skeleton were subjected to direct comparison of TRAP staining to detect bone resorption. Both decalcified, paraffin-embedded tissues and undecalcified, cryo-embedded tissues were developed at 37 °C. In both tissues, TRAP-positive osteoclasts lined the vascular spaces throughout the alveolar bone (Fig. 3I,J) with the only difference being that staining in paraffin-sections took up to ~ 3 h while staining in cryosections took ~ 10 min.

Alkaline phosphatase activity was used to detect mineralization by osteoblasts and again, undecalcified, cryo-embedded tissues stained faster and showed a sharper boundary between positive and negative tissues than did decalcified, paraffin-embedded tissues (Fig. 3K,L). Staining of paraffin-embedded tissues was developed at room temperature overnight. The same staining protocol was used for undecalcified, cryo-embedded tissues, however, staining was developed in ~ 1–5 min.

Protein antigenicity was also preserved in undecalcified, cryo-embedded tissues. For example, PCNA, marker of cell proliferation, was detected in both cryo-embedded and paraffin-embedded tissues (Fig. 3M,N). Osterix is a transcription factor expressed by osteoprogenitor cells and here, immunostaining was more sensitive in cryo- versus paraffin-embedded samples (compare Fig. 3O,P). From these analyses we concluded that in our hands, the undecalcified, cryo-embedded tissues retained enzymatic activity and protein antigenicity better than decalcified, paraffin-embedded tissues we prepared.

### Undecalcified, cryo-embedded tissues enable mineral apposition rate analyses

**Figure 4:**
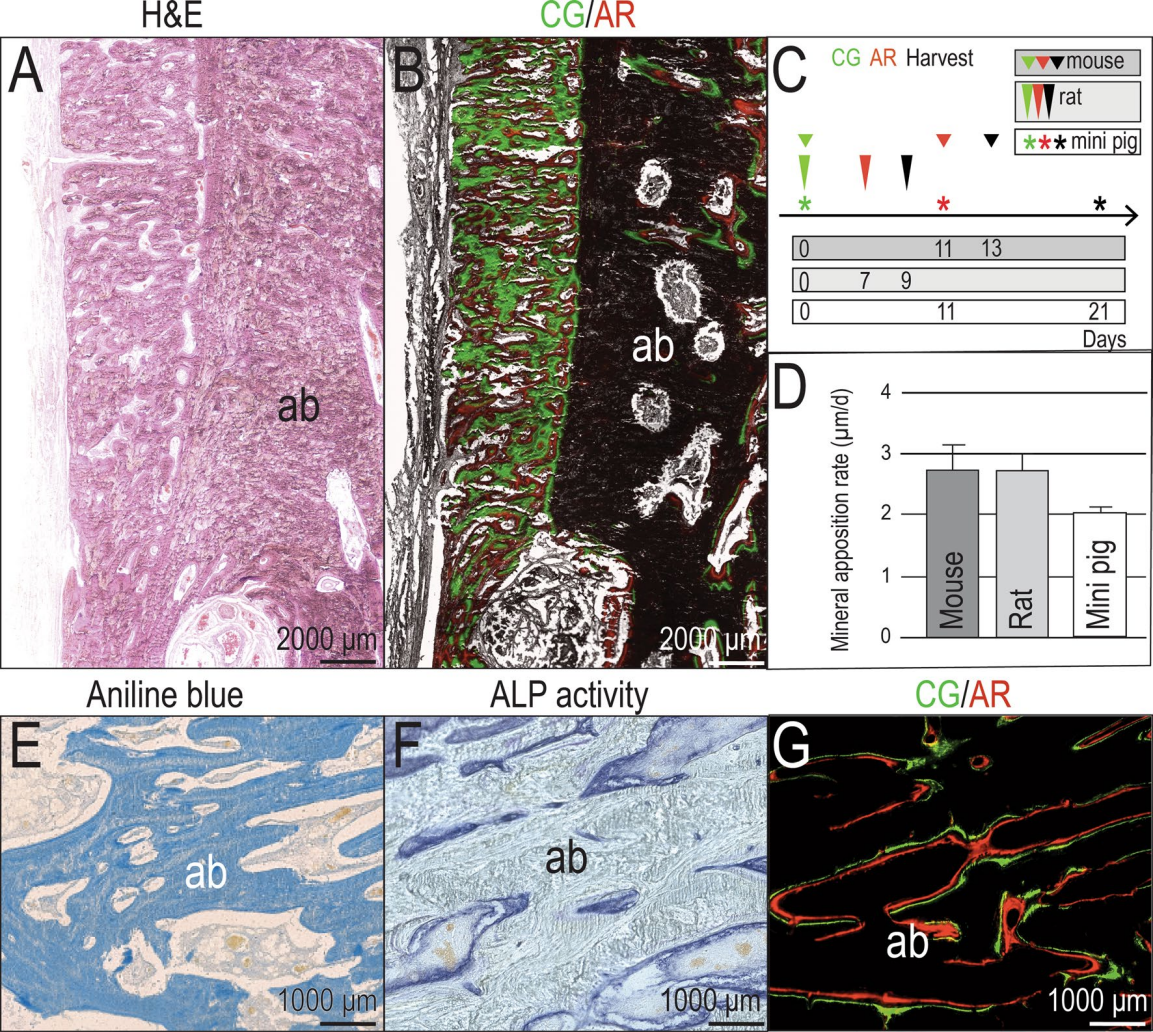
Dynamic bone histomorphometry can be compared across multiple animal models. (**A**) Mini pig buccal bone, transversal section on cryofilm, H&E staining; (**B**) Mini pig buccal bone, transversal section on cryofilm, vital dye labeling; (**C**) Timelines of vital dye labeling in mouse, rat and mini pig; time intervals are as indicated. (**D**) MAR calculations based on the labeling strategies presented in C. Kruskal Wallis test n.s. (**E**) Mini pig alveolar bone, transversal section on cryofilm, Aniline blue staining; (**F**) Mini pig alveolar bone, transversal section on cryofilm, ALP staining; (**G**) Mini pig alveolar bone, transversal section on cryofilm, vital dye labeling detection. Scale bars as indicated. Error bars represent the standard error of the mean. *ab* alveolar bone.

Thus far, our analyses demonstrated that the morphology of, and remodeling activity in, hard tissues was maintained throughout the cryo-embedding and sectioning process, equivalent to that of decalcified, paraffin-embedded hard tissues. We next turned to an analysis which can only be carried out in undecalcified tissues, e.g., vital dye labeling to assess the rate of new bone formation. For example, from an H&E stained section, new bone covering the surface of the mandible and adjacent alveolar bone appeared equivalent (Fig. 4A) but vital dye labeling clearly identified the former as a site of rapid bone accumulation compared to latter (Fig. 4B).

Given the ability to detect vital dye labeling in mini pig samples, we sought to compare whether dynamic histomorphometric analyses such as mineral apposition rate (MAR) in mini pig samples were as robust and reliable as the same types of analyses carried out in rodents e.g., mice and rats. Indeed, using labeling periods appropriate to the species (Fig. 4C), the MAR calculated from analyses of cryo-sectioned mini pig tissues were on par with the MARs calculated for rodents: in mini pigs, the MAR was ~ 2.1 µm/day; in mice and in rats, MARs were ~ 2.7 µm/day (Fig. 4D). Sites in mini pig samples undergoing bone remodeling could be readily identified by this method: for example, compared to Aniline blue stained tissues (Fig. 4E), on a near-adjacent section, ALP stained sites of active mineralization (Fig. 4F), which precisely corresponded with sites of vital dye labeling (Fig. 4G).

## Discussion

Despite significant differences in bone densities and microarchitecture^15,16^, mini pigs continue to be the one of the most commonly used large animal models for testing of orthopedic and dental devices intended for use in humans^17^. Therefore, it struck us that the more information that could be gleaned from studies using a mini pig model, the better would be the translational value of that information. Our goal here was to develop and test a method for cryo-processing that supported the greatest number of quantitative assays, while preserving tissue architecture and shortening the timeline to obtain these data. Mini pig tissue samples were processed via paraffin-embedding versus cryo-embedded to facilitate a rigorous comparison of the methodologies (Fig. 3). These side-by-side comparisons demonstrated that high quality, undecalcified hard tissue sections from a large animal can be generated via cryo-processing, and the quality of these are on par with paraffin-processed sections from the same species.

Given the hardness of the bone, resin-embedding is the most common method of tissue processing^18,19^. While the resin produces a uniformly hard material for cutting and the technique produces tissue sections with very good cell morphology, the entire procedure is destructive. The process involves toxic chemicals which harden via an exothermic reaction and the resulting heat is detrimental to antigen preservation^20^ and thus histochemical and immunohistochemical analyses. Such analyses allow researchers to identify areas undergoing active bone formation and resorption and pinpoint the location of proteins of interest in the skeleton. Since these assays cannot be readily performed on resin-embedded tissues, investigators must decide whether to increase the number of animals in a study and allocate a subset of these animals for methods of tissue processing other than resin-embedding- or they must forgo these analyses altogether and thus lose potential information that may be of considerable value in device and/or drug development. Resin-embedding is also time-consuming e.g., even in small bone specimens it takes several weeks to complete processing and sectioning^21^. Each resin-embedded specimen typically yields a single tissue section for analysis, which represents a significant limitation of the method.

Another strategy for analyzing hard tissues from large animals is to make the tissue uniformly soft by removing mineral via decalcification, followed by paraffin-embedding. However, paraffin-processed tissues are not amenable to vital dye labeling/MAR measurements because decalcification of the bone tissue removes the mineral component of bone, and any mineral labeling^22^. Decalcification is carried out using reagents that chelate calcium^20^ and while decalcification with strong acids such as hydrochloric acid is rapid (on the order of 12–24 h), the procedure adversely affects cell morphology^23^ and degrades proteins, resulting in poor and/or ineffective immunohistochemical staining^24^. Using a milder chelating agent for decalcification such as EDTA preserves tissue morphology^23^ but decalcification proceeds slowly, with complete decalcification of mineralized tissues from large animals taking several months^25^. In our experience with mini pig mandibles, complete decalcification took 7 months.

An alternative to resin- and paraffin-processing is cryo-processing. Relative to other embedding methods, we found cryo-embedding to be fast e.g., from fresh tissue to a block ready to section takes ~ 19 days. Cryo-sectioning can also yield hundreds of sections that are amenable to the vast majority of analytical assays. If freezing artifacts are avoided, tissue morphology and microarchitecture are well-preserved. Cryo-processing preserves cellular/molecular integrity and because decalcification is avoided, calcium-dependent assays including MAR can be simultaneously readily assessed in the processed tissues. Therefore, the number of animals required for a given study may be reduced and/or more information can be gleaned from each animal study.

The major disadvantage associated with cryo-preservation of undecalcified hard tissues from large animals is the technique-sensitive nature of the process. Consequently, most cryo-processed hard tissues from large animals are first decalcified^26^. Cryo-processing does work well for small hard tissue samples e.g., from rodents^27–30^ but here our interest was in hard tissues from large animal studies that are typically performed for preclinical safety/toxicity testing of drugs and devices intended for use in humans.

Methods for handling undecalcified resin-embedded tissue sections on adhesive tapes were first proposed 30 years ago^31^. Since then, several tape techniques for handling cryo-embedded tissues have been introduced^32,33^ but obtaining high-quality undecalcified sections has consistently been described as difficult^34^. To our knowledge, this is the first report of cryo-embedding tape techniques for the analysis of undecalcified large bone specimens. By using step-wise dilution and gradual infiltration of the cryo-embedding media (see Methods), we circumvented problems associated with tissue destruction/loss of morphology. Compared to resin-embedded samples that typically yield very few tissue sections, cryo-embedded samples yielded hundreds of high-quality tissue sections. As a consequence, more histochemical/immunohistochemical analyses can be carried out on tissues from each animal. This information, in turn, can provide a deeper understanding of three-dimensional tissue architecture, as well as the tissue-level responses to devices and drugs.

We found that in cases where antibodies had not been validated for use in pigs, it was still possible to generate tissue sections with specific immunostaining patterns (Fig. 3). We show enzyme activity and protein antigenicity superior to decalcified, paraffin-embedded tissues, which indicates higher sensitivity in cryo-embedded tissues (Fig. 3). Although the use of cryofilm resulted in images with lower sharpness, cryo-embedded tissue architecture was well-preserved.

This study demonstrated a new method for processing tissues from large animals that rendered them amenable to commonly performed molecular and cellular analyses. In turn, this information may be useful when predicting human responses to a drug or device based on large animal studies. In this regard, one variable that is typically overlooked is the alignment between the skeletal age of the patient population benefitting from the drug or device, and the skeletal age of most animals used in preclinical studies. Animals are typically young and, in most cases, have not even reached peak bone mass^35,36^. For example, in mice, peak bone mass is reached at ~ 4 months of age; in rats at ~ 9 months of age and in mini pigs, between ~ 2 and 3 years^37–39^. While it would be an obvious advantage to use animals whose skeletal age aligned with the intended patient population, this is very rarely achieved because the cost associated with housing large animals until they have reached peak bone mass (or older) is prohibitive.

An independent, non-governmental, international organization i.e., the International Organization for Standardization, the ISO, publishes specifications that are used to evaluate and compare products, materials, services, and processes. In maxillofacial surgery and implant dentistry related research, for example, ISO guidelines indicate that pivotal preclinical testing should be carried out in an animal model with jaws of sufficient size to accommodate the medical device e.g., dental implant being tested. The animal models should also have an opposing dentition and non-herbivorous pattern of masticatory jaw movement (ISO 7405:2018). The pig—and more specifically, the mini pig—is therefore considered by ISO guidelines to be a suitable animal model for testing dental implant systems (see^40^ for a recent review on the use of pigs for animal research).

We find, however, that the vast majority of preclinical studies in mini pigs have very small sample sizes. The high costs associated with purchase and housing of large animals, along with the duration of the study, are critical factors in this regard^41^. This is especially relevant when evaluating clinical conditions that are slowly progressive/degenerative in nature^42^; such studies are often cost-prohibitive in large animals. The cryo-processing methods shown here are readily adaptable to tissues from human subjects and other large animal models, on par with that typically found in rodent studies. By rendering mini pig tissues amenable to histochemical/immunohistochemical analyses typically reserved for rodents, it may be possible to extract more information from preclinical studies, which can in turn help ensure the safety and efficacy of a device or drug as required by regulatory bodies.

There are sound ethical arguments for reducing, wherever possible, the number of animals used in laboratory testing^43,44^. Since neither in silico nor in vitro methods/models fully recapitulate the process of tissue healing, the most logical means to reduce animal usage is to minimize numbers while simultaneously maximizing the amount of information we learn from each animal^45^. This would have enormous potential benefit, not only by gaining more insights from any given preclinical study, but also by limiting the number of animals that must be sacrificed to support development of drugs and devices intended for use in humans.

## Supplementary information

Supplementary Information 1.

## Data Availability

The datasets generated during and/or analyzed for the current study are available from the corresponding author.
