# Supplementary material for: A novel cryo-embedding method for in-depth analysis of craniofacial mini pig bone specimens
Source: Sci Rep. 2020 Nov 11;10:19510. doi: 10.1038/s41598-020-76336-3 (PMC7658236; doi:10.1038/s41598-020-76336-3)
Supplement: Supplementary file 1 — Supplementary Information 1. [file 41598_2020_76336_MOESM1_ESM.pdf]

A NOVEL CRYO-EMBEDDING METHOD FOR IN-DEPTH ANALYSIS OF CRANIOFACIAL MINI PIG BONE SPECIMENS

Pavla Ticha, MD, MA[1,2]; Igor Pilawski, DVM, PhD[1]; Xue Yuan, PhD[1]; Jie Pan, DDS[1]; Ustun S. Tulu, PhD[1]; Benjamin R. Coyac, DDS, PhD[1]; Waldemar Hoffmann, PhD[3], and Jill A. Helms, DDS, PhD[1]*

[1]Division of Plastic and Reconstructive Surgery, Department of Surgery, Stanford University School of Medicine, Stanford, CA 94305, USA

[2]Clinic of Plastic Surgery, 3rd Faculty of Medicine and University Hospital Kralovske Vinohrady, Charles University in Prague, Srobarova 50, 10034, Praha 10, Czech Republic

[3]Nobel Biocare Services AG P.O. Box, CH-8058 Zürich-Flughafen, Switzerland

* Corresponding author:

Jill A. Helms, Stanford University, 1651 Page Mill Road, Palo Alto, CA 94304, USA

E-mail: jhelms@stanford.edu

Supplementary Information

Mini pig handling

After a minimum of 7 days' acclimation, a physical examination of animals was performed under the supervision of the veterinarian. Animals were individually housed in enclosures that conformed to standards set forth in the Guide for the Care and Use of Laboratory Animals (National Research Council (US) Committee, Washington (DC): National Academies Press (US); 2011). A soft-food diet was used throughout the study and water was provided *ad libitum.*

Vital dye administration in mice and rats

Alveolar bone samples from 5 BALB/c mice (1.5 months old females) were used. Mice were housed at Stanford University in Stanford, CA and the ethical protocol was reviewed and approved by Stanford University Committee on animal welfare. Animals were administrated intraperitoneally with calcein green (25 mg/kg, dissolved in 0.15 M NaCl and 2% $NaHCO_3$) and alizarin red (25 mg/kg, dissolved in saline solution). The time interval between fluorochrome injections was 11 days. Two days post-alizarin red injection, euthanasia of mice was performed by carbon dioxide asphyxiation. Cryo-embedding and sectioning were performed as described.

Alveolar bone samples from 8 Sprague-Dawley rats (3 months old females) were used. Rats were housed at Stanford University in Stanford, CA and the ethical protocol was reviewed and approved by Stanford University Committee on animal welfare. Animals were injected intraperitoneally with calcein green (25 mg/kg, dissolved in 0.15 M NaCl and 2% $NaHCO_3$) and alizarin red (25 mg/kg, dissolved in saline solution). The time interval between fluorochrome injections was 7 days. Two days after alizarin red injection, rats were sacrificed by carbon dioxide asphyxiation. Cryo-embedding and sectioning were performed as described.

Mini pig sacrifice, tissue collection and fixation

Ten days after alizarin red administration, mini pigs were euthanized by a rapid bolus injection of pentobarbital (80-120 mg/kg). Death was confirmed by auscultation. Mandibles were then divested of most of the muscles, tendons, ligaments and crowns of the teeth and each mandible was divided into two parts by a rotating saw. Minimal soft tissues including the gingiva and some muscle tissues were preserved on each side of hemi-mandible. Tissues were rinsed in sterile ice-cold phosphate-buffered saline (PBS) to remove debris and blood and then transferred into 4% paraformaldehyde (PFA). The volume of PFA fixative was ~5 times the volume of the tissue block

itself. Then PFA fixation was performed at 4 °C on a rocking platform protected from light for 72 h. Thereafter, tissues were rinsed and transferred back into PBS where they have been stored until dissection.

Mice and rats tissue processing

Mouse and rat alveolar bone tissues were dissected and fixed in 4% PFA at 4 °C overnight. Tissues were then washed in PBS. Thereafter, tissues were cryopreserved in 30% sucrose - mouse samples for 12 h, rat samples for 7 days. Alveolar bone samples were then directly cryo-embedded via mixture of polyethylene oxide (PEO) and carboxymethyl cellulose (CMC), cryo-sectioned and collected on an adhesive film as described in the main text, using disposable microtome blades (#30-538-35, Ultra Disposable Microtome Blades, Thermo Fisher Scientific, Waltham, MA, USA).

Vital dye detection, image acquisition and quantification

Mineral apposition rate (MAR) represents the rate at which osteoid matrix, a product of osteoblasts, is deposited and mineralized. Mini pig, rat and mouse tissues labeled *in vivo* with vital dyes are amenable to MAR measurement with quantification of site-specific changes in bone formation.

Calcein green and alizarin red labeling was detected on the sections collected with cryofilm as described above. Image acquisition was performed by means of fluorescence microscopy (Leica DMi8 with DMC4500 digital camera, Leica Camera AG, Germany). Adobe Photoshop (Adobe Inc., San Jose, CA, USA) was utilized to create a superimposed image consisting of a greyscale brightfield picture showcasing the respective morphology as well as both the calcein green and alizarin red labeled areas within the same ROI.

Using ImageJ software (NIH, Bethesda, Maryland, USA) the distance between the alizarin and calcein labeled bone was quantified. Per section, a minimum of 15 measurements were performed. The width of newly mineralized osteoid matrix was quantified by measuring the span between two parallel fluorescent labels in designated samples.

Statistical analyses

Results are presented as the mean ± standard error of the mean of independent replicates ($n \geq 3$). The non-parametric data, as determined by D'Agostino & Pearson normality test, was analyzed by Kruskal Wallis test. $P \leq 0.05$ was considered significant. GraphPad Prism v. 7 (GraphPad Software, Inc., San Diego, USA) was used for these analyses.

Histological staining of undecalcified, cryo-embedded tissue sections

Hematoxylin & eosin staining worked on tissue sections that were collected with cryofilm as well as sections collected with transfer film. Tissue sections on adhesive film were stained by 5 drops

of Carazzi's Hematoxylin solution. After 1 min, the adhesive film with the tissue section was positioned in a Coplin jar and washed in tap water for 4 min. Thereafter, 5 drops of 0.2% Eosin Y solution were applied. After 10 s, the film was washed briefly in tap water and rinsed in 100% ethanol for 10 s to partially dehydrate the tissues. Sections on cryofilm were then covered with a water-based mounting medium consisting of 30% glycerin (#C-MM001, SCMM-G1, Section lab, Hiroshima, Japan) and sandwiched between the film and a glass slide. Sections on transfer film were transferred to a glass slide (see in the main text) and cover-slipped with xylene-based mounting medium Permount.

Aniline blue staining worked on tissue sections that were collected with cryofilm as well as sections collected with transfer film. Tissue sections were stained in a saturated solution of picric acid for 30 s, washed in tap water for 10 min and rinsed in double distilled water (ddH_2O). Thereafter, sections were immersed in 5% phosphotungstic acid-5% phosphomolybdic acid solution for 15 min, followed by staining with 1% Aniline blue solution for 10 min. Afterwards, sections were differentiated in a solution of 1% acetic acid and dehydrated using a graded ethanol series to Citrisolv, each step for 30 s. Sections on cryofilm were then covered with a water-based mounting medium and sandwiched between the film and a glass slide, sections on transfer film were transferred to glass slide and cover-slipped with xylene-based mounting medium.

Masson's trichrome staining worked on tissue sections that were collected with cryofilm as well as sections collected with transfer film. Sections were placed in a container with Bouin's fixative covered with aluminum foil. After 1 h at 56 °C, the solution was allowed to cool, and sections were washed in tap water to remove excess yellow stain. Thereafter, sections were stained with Weigert's iron hematoxylin for 5 min, washed with tap water for another 10 min and rinsed in ddH_2O. Sections were then placed in Biebrich scarlet-acid fuchsin for 15 min, rinsed in ddH_2O, treated by 5% phosphotungstic acid-5% phosphomolybdic acid solution for 15 min followed by staining with 1% Aniline blue solution for another 10 min. Thereafter, sections were rinsed in ddH_2O, differentiated in 1% acetic acid for 3-5 min and dehydrated using a graded ethanol series to Citrisolv, each step for 30 s. Sections on cryofilm were then covered with a water-based mounting medium and sandwiched between the film and a glass slide. Sections on transfer film were transferred to glass slide and cover-slipped with xylene-based mounting medium.

Movat's pentachrome staining worked on tissue sections that were collected with cryofilm as well as sections collected with transfer film. Tissue sections were stained in 1% alcian blue for 10 min, washed in tap water for 10 min and rinsed in ddH_2O. Thereafter, sections were stained for 5 min with the following mixture: solution 1 containing 1% Verhoeff's hematoxylin dissolved in absolute ethanol was prepared; solution 2 containing ~1.16% ferro-III-chloride and 0.37% saturated HCl

was prepared; then, solution 1 and solution 2 were mixed by 1:1 ratio. Thereafter, sections were rinsed in ddH_2O and washed in tap water for 5 min, immersed in solution containing 7.85% Sodium Thiosulfate for 1 min and washed in tap water for another 5 min. Then, sections were treated with a fresh solution containing 0.08% Crocein-Scarlet, 0.02% Acid Fuchsin, both dissolved in 1% glacial acetic acid and ddH_2O for 8 min. Afterwards sections were briefly rinsed in ddH_2O followed by fresh solution of 0.5% acetic acid and immersed in 5% phosphotungstic acid for 15 min. Then, sections were differentiated in solution of 0.5% acetic acid for 1 min and immersed in 100% ethanol for 5 min three times followed by staining in saffron for 60 min. Sections were dehydrated using a graded ethanol series to Citrisolv, each step for 30 s. Finally, sections on cryofilm were covered with a water-based mounting medium and sandwiched between the film and a glass slide, sections on transfer film were transferred to a glass slide and cover-slipped with xylene-based mounting medium.

Picrosirius red staining only worked on tissue sections that were collected with transfer film. Tissue sections on transfer film were stained with 0.1% sirius red dissolved in a saturated picric acid for 60 min to discriminate tightly packed and aligned collagen molecules. Thereafter, sections were washed for 5 min in 1% acetic acid twice, then rinsed in ddH_2O. Tissue sections were transferred to glass slides and cover-slipped with xylene-based mounting medium.

Histochemistry of undecalcified, cryo-embedded tissue sections

Prior to histochemical staining, samples on cryofilm were washed in PBS for 10 s.

TRAP activity detection was performed on tissue sections collected with cryofilm. Sections were put into a wet chamber, each section was applied with 500 µL of TRAP staining solution (#387A-1KT, Acid Phosphatase, Leukocyte (TRAP) Kit, Sigma Aldrich, St. Louis, MO, USA) and incubated at 37 °C. TRAP staining developed in 10 min. Then the sections were washed with PBS and mounted with water-based mounting medium between adhesive film and glass slide.

ALP activity detection was performed on tissue sections collected with cryofilm. Each section was applied with 500 µL of NTMT solution (mixture of 0.25 mL of 5 M $MgCl_2$; 0.2 mL of 5 M NaCl; 10 µL of Tween 20 and 1 mL of 1 M Tris, pH 9) and incubated at room temperature for 30 min. Then the sections were incubated with 500 µL of ALP staining solution for each section (#34042, 1-Step NBT/BCIP Substrate Solution, Thermo Fisher Scientific, Waltham, MA, USA) at room temperature for 1-5 min, until the color reaction was complete. Afterwards, sections were washed with PBS and mounted with water-based mounting medium between adhesive film and glass slide.

Immunohistochemistry of undecalcified, cryo-embedded tissue sections

Immunohistochemical staining was performed on tissue sections collected with cryofilm. In brief, sections were washed in PBS, permeabilized using 0.5% Triton X-100 for 5 min with gentle shaking and washed for 5 min with PBS twice with shaking. Then, each section was applied with 200-300 µL of 1% antigen unmasking solution (#H-3300, Antigen Unmasking Solution, Citric Acid Based, Vector Laboratories, Burlingame, USA) and incubated in a moist chamber at 95-98 °C for 30 min. After sections were cooled to room temperature, they were washed for 5 min with PBS twice with shaking. Endogenous peroxidase activity was blocked with 3% hydrogen peroxide for 5 min and washed for 3 min with PBS three times. Sections were then blocked with RTU Animal-Free diluent (#SP-5035, RTU Animal-Free Blocker® and Diluent (AFB), Vector Laboratories, Burlingame, CA, USA) at room temperature for 30-60 min. Thereafter, the appropriate primary antibody was diluted in RTU diluent (at dilutions indicated below) and 200-300 µL of the solution was applied on each section. Sections were incubated at 4 °C overnight, then washed for 5 min with PBS four times. Sections were then incubated with appropriate biotinylated secondary antibodies, (#BA-1000, Biotinylated Goat Anti-Rabbit IgG antibody, Vector Laboratories, Burlingame, CA, USA) diluted to 1:200 with RTU at room temperature for 30 min and washed for 5 min with PBS four times. An avidin/biotinylated enzyme complex (#32050, Ultra-Sensitive ABC Peroxidase Standard Staining Kit, Thermo Fisher Scientific, Waltham, MA, USA) was added, sections were incubated at room temperature for 30–60 min and then washed with PBS three times with gentle shaking. DAB reagent (#SK-4100, DAB kit, Vector Laboratories, Burlingame, CA, USA) was used to develop the color reaction, which took 20 s–10 min. Sections were rinsed in tap water for 1 min and mounted with water-based mounting medium between adhesive film and glass slide. Primary antibodies included PCNA (#ab29, Abcam; 1:5000) and Osterix (#ab225522, Abcam; 1:100)

Image acquisition of undecalcified, cryo-embedded, stained tissue sections

Image acquisition of tissue sections mounted between cryofilm and glass slide was performed by means of brightfield microscopy with higher light intensity to avoid cryofilm artefacts. Imaging of tissue sections transferred to glass slide was performed by means of DIC microscopy (Leica DMi8 with Leica DMC4500 digital camera, Leica Camera AG, Germany). Image acquisition of tissue sections stained with Picrosirius red was performed by means polarized light microscopy.

Focus stacking (Z-stacking) was performed to achieve an extended depth of field, which was of particular importance in 20x and 40x images. As the digital camera mounted onto the microscope exhibited only a limited dynamic range, high dynamic range imaging (HDR) was applied in order to acquire a similar range of luminance that was experienced when looking at the tissue section using the human eye. HDR was achieved by acquiring a set of differently exposed images of a given

sample that were then digitally merged with LAS X Software (Version 3.0.16120.2, Leica Camera AG, Germany) to produce the final HDR image.

Decalcification, paraffin-embedding and –sectioning

Part of the tissue specimens were decalcified and paraffin-embedded. To decalcify the tissues, each specimen was kept in 19% EDTA solution at room temperature for 7 months on a shaking table. EDTA was changed three times a week. The degree of decalcification was tested by insertion of a needle. After demineralization, specimens were dehydrated through an ascending ethanol series, e.g., 50%, 75%, 85%, 95% and finally two times 100% ethanol. Each step was undertaken at 4 °C overnight to ensure complete dehydration. For the paraffin-embedding, specimens were cleared in xylene for 15 min, then infiltrated with a mixture of xylene and paraffin (1:1) for 1 h followed by 100% paraffin for 48 h three times. Each paraffin step was performed at 60 °C under vacuum. Tissue sections were generated at an 8 µm thickness and collected on glass slides.

Histological staining of decalcified, paraffin-embedded tissue sections

Prior to staining, tissue sections were dewaxed and hydrated through a descending grade ethanol series. For staining of decalcified, paraffin-embedded tissues, the same protocols were followed as for cryo-embedded tissues. However, some procedures, e.g., staining and incubation times, required adjustments. The differences are described below. All stained paraffin-embedded sections were then dehydrated in an ascending ethanol series followed by Citrisolv and cover-slipped with Permount.

Paraffin-embedded sections were stained with Aniline blue according to the protocol for cryo-embedded sections, however, time of staining with 1% Aniline blue solution was reduced from 10 min to 1-5 min.

In paraffin-embedded sections, time of Movat's pentachrome staining with 1% alcian blue was reduced from 20 to 10 min. Thereafter, the same protocol was employed as in cryo-embedded sections.

Histochemistry of decalcified, paraffin-embedded tissue sections

TRAP staining was performed as previously described, however, the incubation period with TRAP staining solution (#387A-1KT, Acid Phosphatase, Leukocyte (TRAP) Kit, Sigma Aldrich, St. Louis, MO, USA) required an extension from 10 min to ~3 h.

ALP staining was also performed as described above, except for the incubation time with ALP staining solution (#34042, 1-Step NBT/BCIP Substrate Solution, Thermo Fisher Scientific, Waltham, MA, USA), which was extended from 1-5 min to overnight.
